# New Insights into the Liver–Visceral Adipose Axis During Hepatic Resection and Liver Transplantation

**DOI:** 10.3390/cells8091100

**Published:** 2019-09-18

**Authors:** María Eugenia Cornide-Petronio, Mónica B. Jiménez-Castro, Jordi Gracia-Sancho, Carmen Peralta

**Affiliations:** 1 Institut d’Investigacions Biomèdiques August Pi I Sunyer (IDIBAPS), 08036 Barcelona, Spain; cornide@clinic.cat (M.E.C.-P.); monicabjimenez@hotmail.com (M.B.J.-C.); 2 Liver Vascular Biology Research Group, Barcelona Hepatic Hemodynamic Laboratory IDIBAPS, 08036 Barcelona, Spain; jgracia@clinic.cat; 3 Centro de Investigación Biomédica en Red de Enfermedades Hepáticas y Digestivas (CIBERehd), 08036 Barcelona, Spain

**Keywords:** adipose tissue, liver, inflammation, steatosis, liver resection, liver transplantation, lipectomy

## Abstract

In the last decade, adipose tissue has emerged as an endocrine organ with a key role in energy homeostasis. In addition, there is close crosstalk between the adipose tissue and the liver, since pro- and anti-inflammatory substances produced at the visceral adipose tissue level directly target the liver through the portal vein. During surgical procedures, including hepatic resection and liver transplantation, ischemia–reperfusion injury induces damage and regenerative failure. It has been suggested that adipose tissue is associated with both pathological or, on the contrary, with protective effects on damage and regenerative response after liver surgery. The present review aims to summarize the current knowledge on the crosstalk between the adipose tissue and the liver during liver surgery. Therapeutic strategies as well as the clinical and scientific controversies in this field are discussed. The different experimental models, such as lipectomy, to evaluate the role of adipose tissue in both steatotic and nonsteatotic livers undergoing surgery, are described. Such information may be useful for the establishment of protective strategies aimed at regulating the liver–visceral adipose tissue axis and improving the postoperative outcomes in clinical liver surgery.

## 1. Introduction

In the last decade, adipose tissue has emerged as an essential and highly active metabolic and endocrine organ [1,2,3]. The basic function of adipocytes is to take up free fatty acids (FFA) from circulating lipoprotein complexes and esterify them into triacylglycerides [4]. During times of metabolic demand, hydrolysis of triacylglyceride releases FFA to generate adenosine triphosphate (ATP) [5]. These adipocyte processes, termed lipogenesis and lipolysis, respectively, are primarily governed through hormonal pathways [6]. However, one of the most important characteristics of adipose tissue is its function in whole-body energy homeostasis, mediated principally through the endocrine system [4]. Adipose tissue expresses and secretes a variety of bioactive molecules, known as adipokines, which may exert their effects in adipose tissue and in other organs [7]. Adipokines include leptin, interleukin (IL)-6, other cytokines, adiponectin, complement components, adipsin, plasminogen activator inhibitor-1 (PAI-1), and proteins of the renin–angiotensin system, among others [7]. Collectively, adipokines modulate the crosstalk between adipose tissue and other metabolic organs, including the liver [8]. Thus, adipokines directly target the liver through the portal vein [9] and have significant effects on liver diseases [4].

**Figure 1:**
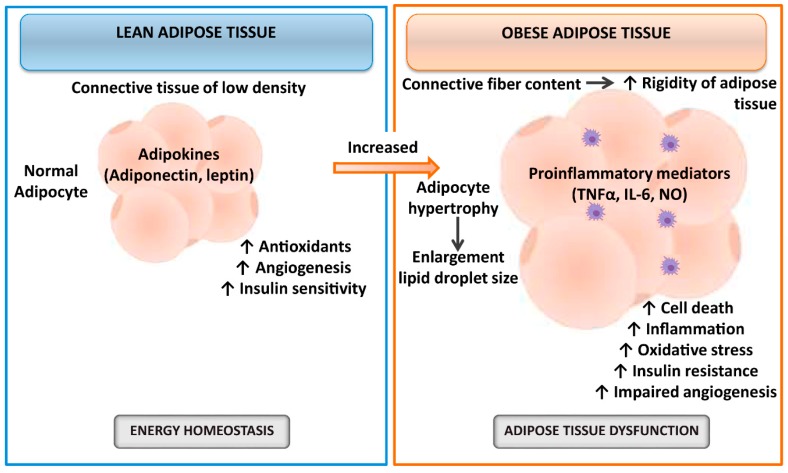
Schematic illustration of functional differences between lean and obese adipose tissue. Abbreviations: IL, interleukin; NO, nitric oxide; TNFα, tumor necrosis factor α.

The hypoxia and subsequent oxygen delivery restoration to the liver, namely, hepatic ischemia–reperfusion (I/R), is one of the major pathophysiological events and causes of morbidity and mortality in liver resections and transplantation, being more evident in the presence of hepatic steatosis [10,11,12,13,14]. Despite the attempts to solve this issue, hepatic I/R is an unresolved problem in clinical practice [15]. The cellular mechanisms involved in liver I/R injury are numerous and complicated [14], which led to discrepancies in our understanding of this pathology [16]. For instance, the mechanisms underlying I/R injury in conditions of cold ischemia associated with liver transplantation (LT) are different from those that occur in conditions of warm ischemia associated with liver resections. In addition, hepatic steatosis is associated with an increased postoperative complication index and mortality after liver resection and transplantation, and the mechanisms responsible for hepatic damage and regenerative failure are different in steatotic versus nonsteatotic livers [15]. The investigations focused on the role of adipose tissue are of clinical and scientific relevance since the prevalence of obesity ranges from 24–45% of the population and consequently is expected to increase the number of steatotic livers submitted to surgery, which poorly tolerate I/R damage, resulting in liver dysfunction and regenerative failure [17,18,19,20,21,22,23]. In addition, it has been reported that adipose tissue exerts both pathological or, on the contrary, protective effects on damage and regenerative response [24]. It should be noted that functional differences between lean and obese adipose tissue have been extensively described [25,26,27] and summarized, as seen in Figure 1. Briefly, adipose tissue from lean individuals is a connective tissue of low density with small insulin-sensitive adipocytes that secrete adipokines involved in energy homeostasis, angiogenesis, and antioxidant processes. However, the rigidity of adipose tissue from obese individuals is caused by the increment of connective fiber content. Hypertrophic insulin-resistant adipocytes secrete different inflammatory mediators, resulting in adipose tissue dysfunction, impaired angiogenesis, and cell death [25,26,27]. Moreover, obesity induces changes in the secretion of adipokines from adipose tissue to the circulation [28,29,30] and increases the inflammatory response and oxidative stress in adipose tissue [31,32,33,34,35]. Therefore, investigations focused on evaluating the liver–adipose tissue axis in steatotic and nonsteatotic livers subjected to hepatic resections or transplants are highly useful in the establishment of specific therapies to prevent both hepatic I/R injury and regenerative failure in liver surgery.

In the first part of this review, we highlight the actual knowledge of the crosstalk between the adipose tissue and the liver during liver surgery. In addition, the different experimental models and pharmacological strategies aimed at regulating potential dysfunctions in the adipose tissue–liver axis in liver surgery are presented, focusing on the strengths and limitations. Clinical results on the role of adipose tissue in the postoperative outcomes after liver surgery are also discussed.

## 2. Relevance of Adipose Tissue in Experimental Models of Liver Resection

Crosstalk between the adipose tissue and the liver is an important event both in the physiological function of the liver and in the development of liver diseases [4]. Indeed, adipose tissue inflammation is a well-recognized sign of obesity [36,37], one of its major consequences being the alteration of the secretion of adipokines that drain to the liver via the portal vein, a notion known as the “portal theory” [37,38,39]. This dysfunctional adipose tissue–liver axis is supported by the specific disruption in adipocytes of inflammatory mediators (apoptosis antigen 1 or cluster of differentiation 95; Fas/CD95) and/or inflammatory signals (c-Jun N-terminal kinase-1, JNK1) in different mouse models, resulting in protection against hepatic steatosis [40,41].

**Figure 2:**
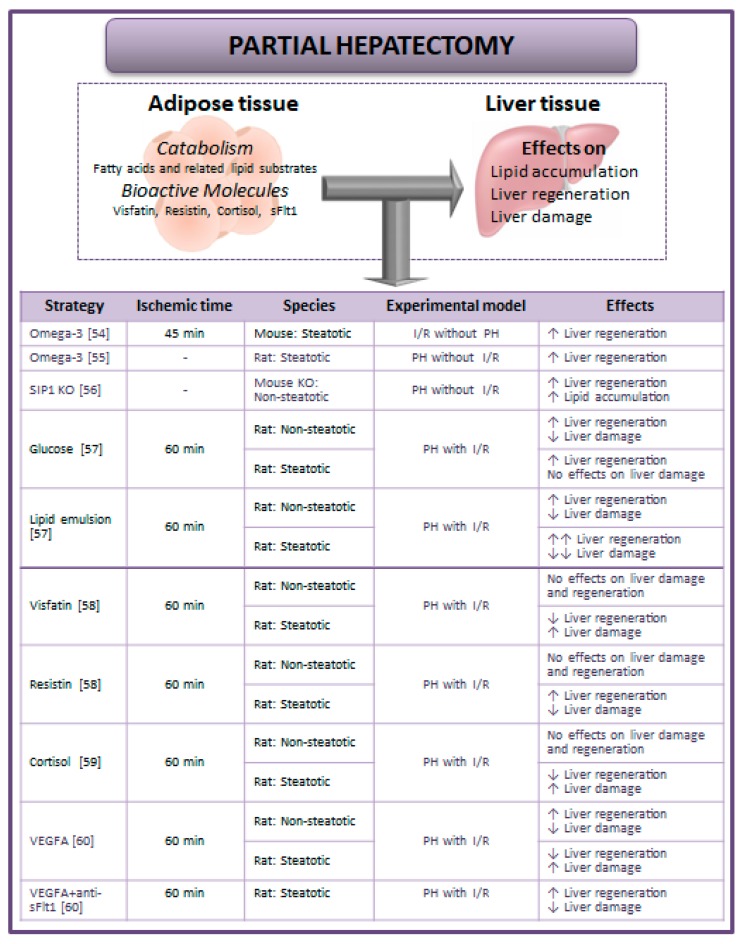
Strategies aimed at regulating hepatic damage and regenerative failure considering the adipose tissue–liver crosstalk during partial hepatectomy (PH) with or without I/R [54,55,56,57,58,59,60]. Abbreviations: I/R, ischemia–reperfusion; PH, partial hepatectomy; sFlt1, soluble form of the VEGF receptor 1; SIP1, smad interacting protein 1; VEGFA, vascular endothelial growth factor type A.

It has been suggested that lipids are the preferred energy substrate for nonsteatotic livers in conditions of partial hepatectomy (PH) without I/R [42,43,44]. Briefly, hypoglycemia that follows PH induces catabolism of peripheral adipose stores followed by hepatic accumulation of systemically derived fat and subsequent liver regeneration [45,46,47]. This is supported by the fact that glucose administration could block the mobilization of fatty acids from adipose tissue by the liver to obtain energy [48]. Parameters of lipid metabolism have been reported during hepatic regeneration: esterification rate of fatty acids from adipose tissue is higher and lipogenesis is raised [49,50]. In fact, the remaining liver after PH expresses the lipoprotein lipase, which could take up fatty acids from circulating triacylglycerides [51]. Moreover, different studies have described that mice lacking lipid metabolism-associated genes have reduced hepatic adipogenesis and regeneration liver failure [52,53]. In line with this, the surgical relevance of the lipid lowering effect of omega-3 fatty acids has been studied in steatotic livers in the setting of hepatic I/R injury without PH [54] or in PH without I/R [55] (Figure 2). It should be noted that the process of liver regeneration requires careful regulation of lipid accumulation. In fact, Yang et al. suggested that Smad interacting protein-1 (SIP1) (a key factor linked to the transforming growth factor-β (TGF-β), bone morphogenetic protein (BMP), and Wnt signaling pathways) is one of the mechanisms involved in the hepatic lipid accumulation and, consequently, in the process of liver regeneration under PH without I/R [56] (Figure 2). It is well known that in order for the liver to regenerate, the provision of fatty acids, phospholipids, and cholesterol to the liver is essential for the maintenance of the rate of formation of the membranes of dividing liver cells [50,51]. On the other hand, important questions for steatotic livers arise from these data since further studies will be required to elucidate whether steatosis may be reduced to avoid the vulnerability of steatotic livers to I/R and the regenerative failure, or instead if drugs aimed at increasing the levels of hepatic triglycerides should be used during surgery and thus conserve the energy required for liver regeneration.

The studies mentioned above in liver surgery have been reported in settings of I/R without PH or in PH without I/R. However, it should be noted that in the clinical setting, PH is usually performed under vascular occlusion. Thus, if our aim is the establishment of new protective strategies in the clinical setting of hepatic resection, the experimental conditions used at the bench-side should simulate as close as possible the clinical reality. Fortunately, some studies have evaluated the contribution of adipose tissue to liver injury and regeneration in PH with I/R conditions. Firstly, Mendes-Braz et al. demonstrated that the relevance of adipose tissue in hepatic damage and regeneration depends on the type of liver [57]. In this sense, adipose tissue is not required for the regeneration of nonsteatotic livers subjected to PH with I/R. In contrast, it is necessary to promote regeneration and reduce injury in steatotic livers. Taking these data into account, glucose or lipid emulsion was administered in obese and lean animals undergoing PH + I/R. Glucose or lipid treatment in nonsteatotic livers protected against hepatic damage and regenerative failure. In obese animals, glucose treatment did not protect steatotic livers against damage but improved their regeneration. However, lipid treatment conferred protection against damage and regenerative failure [57]. Mendes-Braz et al. suggest that in addition to the function of adipose tissue as a lipid precursor for new membrane synthesis, the requirement of systemic adipose stores during regeneration of steatotic livers might be based on the endocrine role of adipose tissue as a source of different adipokines, which are essential signals for liver regeneration [57]. 

In addition to the studies related to the role of adipose tissue as a source of energy substrates or inductors of hepatic lipid accumulation, other studies have investigated the potential contribution of adipose tissue as a source of bioactive molecules such as visfatin, cortisol, and soluble forms of the VEGF receptor 1 (sFlt1).

Elias-Miró et al. found the injurious effects of visfatin in PH with I/R and that steatotic livers were more vulnerable to upregulated visfatin than nonsteatotic livers. The administration of visfatin exacerbated damage and regenerative failure in steatotic livers following PH with I/R. Treatment with resistin maintained low levels of visfatin in steatotic livers by blocking its hepatic reuptake from adipose tissue and consequently prevented the injurious effects of visfatin on hepatic damage and regenerative failure [58] (Figure 2).

In pathologic states, adipose tissue may also secret a range of hormones including cortisol, which may be taken up from the circulation by the liver [1,59]. Cornide-Petronio et al. reported that in instances of PH with I/R, the contributory potential of adipose tissue (as a cortisol source) is dependent on baseline liver status (steatotic versus nonsteatotic livers). In such surgical conditions, the authors found that cortisol levels in adipose tissue and liver were elevated only in obese animals [59]. In addition, cortisol administration under PH with I/R conditions exacerbated hepatic damage and regenerative failure only in obese animals. Indeed, in obese animals, alterations in enzymatic regulation of cortisol metabolism caused cortisol accumulation in steatotic livers, whereas in lean animals, compensatory mechanisms mainly based on the clearance of hepatic cortisol were shown to prevent intrahepatic cortisol and its deleterious effects [59]. 

Interestingly, Bujaldon et al. recently examined the effects of vascular endothelial growth factor type A (VEGFA) on damage and regeneration in steatotic and nonsteatotic livers submitted to PH with I/R. The authors reported that VEGFA levels were decreased in both steatotic and nonsteatotic livers after surgery, but the exogenous VEGFA administrated was only able to reach nonsteatotic livers, reducing the incidence of postoperative complications following surgery. Unexpectedly, the authors found that circulating VEGFA was sequestered by the high circulating levels of the sFlt1 released from adipose tissue, so VEGFA could not reach the steatotic liver to exert its effects, ultimately exacerbating damage and regenerative failure [60]. Thus, the concomitant administration of VEGFA and an antibody against sFlt1 was required to avoid binding of sFlt1 to VEGFA. This was associated with high VEGFA levels in steatotic livers and protection against damage and regenerative failure [60] (Figure 2). 

## 3. Relevance of Adipose Tissue in Experimental Models of Liver Transplantation

**Figure 3:**
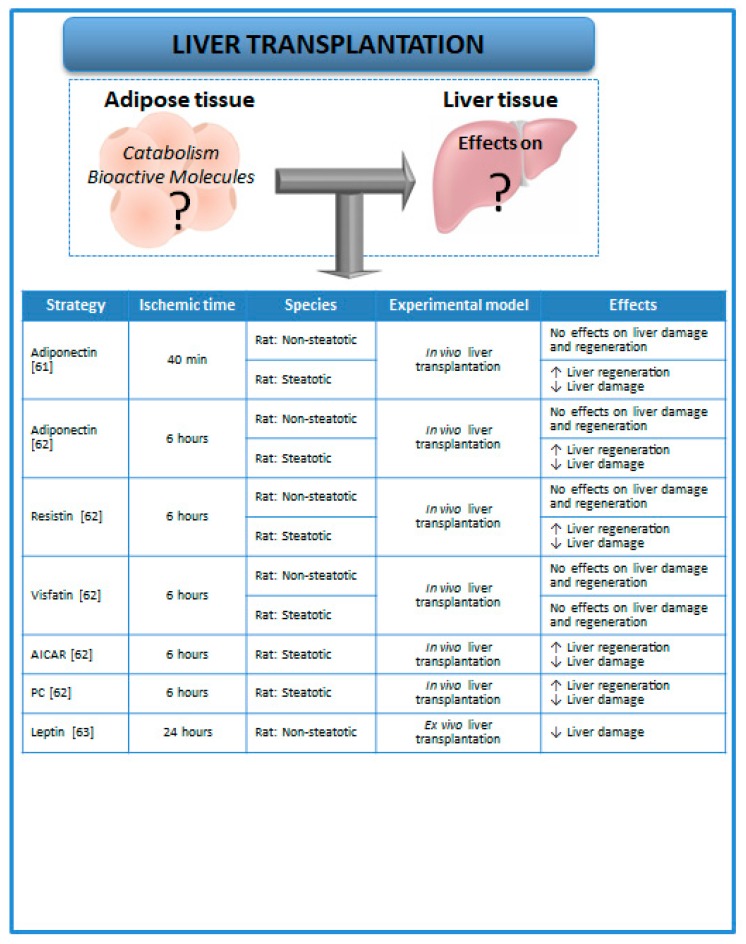
Strategies aimed at evaluating the role of adipokines on hepatic damage and regenerative failure in LT [61,62,63]. The adipose tissue–liver crosstalk during LT is still unknown. Abbreviations: AICAR, Cell-permeable adenosine analog that is a selective activator of AMPK; LT, liver transplantation; PC, preconditioning.

To our knowledge, the relevance of adipose tissue (as a source of fatty acids and related lipid substrates as well as bioactive molecules) on lipid metabolism, hepatic damage, and regeneration associated with transplantation remains to be elucidated (Figure 3). In addition, the few experimental studies on LT [61,62,63] have described the levels of adipokines in the liver but not in adipose tissue. In this vein, it has been demonstrated in experimental studies that adiponectin, resistin, and visfatin levels were not modified in recipients when nonsteatotic livers were subjected to transplantation, whereas in recipients of steatotic liver grafts, the presence of hepatic steatosis down-regulated both adiponectin and resistin levels under such surgical conditions, whereas no changes in visfatin levels were observed [61,62]. The role of adipose tissue as a potential source of adiponectin, resistin, or visfatin was unexplored. Nevertheless, the effects of such bioactive molecules on hepatic damage and regenerative failure were investigated in steatotic and nonsteatotic livers under PH with I/R conditions. As expected, hepatic damage in recipients of steatotic liver grafts was unaltered under pharmacological regulation of visfatin. However, the treatment with either exogenous adiponectin or resistin in steatotic liver grafts improved the postoperative outcomes after transplantation. In addition, the activation of adenosine monophosphate-activated protein kinase (AMPK) by pharmacological drugs such as AICAR (cell-permeable adenosine analog that is a selective activator of AMPK) or ischemic preconditioning (PC)—which increased both adiponectin and resistin in steatotic liver grafts of recipients submitted to transplantation—resulted in protection against hepatic damage [62]. In experimental models of ex vivo LT, it has been reported that the addition of leptin to preservation solutions was able to increase the signal transducer and activator of transcription-3 levels and to reduce damage in nonsteatotic grafts submitted to transplantation [63].

All the studies mentioned above [61,62,63] reported the levels as well as the role of adipokines in liver grafts in experimental models of LT. However, the levels of adipokines in adipose tissue as well as the potential involvement of adipose tissue in the hepatic levels of adipokines following transplantation were not evaluated in such studies. In our view, further investigations to address this issue are of clinical and scientific relevance. In fact, in clinical practice, most of the liver grafts are obtained from brain-dead donors [64]. It is well known that brain death is associated with cerebral trauma and usually caused by hypoxia [65]. In addition, an experimental study aimed at evaluating the role of adipose tissue in the metabolism of rats with brain injury showed that resistin levels were increased in subcutaneous fat of rats with traumatic brain injury [66]. Moreover, it should be taken into account that adipose tissue is considered to be an important source of adipokines, such as leptin, adiponectin, and resistin, and that adipokines directly access the liver from adipose tissue through the portal vein [9,67]. Therefore, the relevance of adipose tissue in the hepatic damage associated with transplantation should be considered in further experimental research of LT to elucidate whether the regulation of adipose tissue functions could improve the quality of donor organs and postoperative outcomes after transplantation.

Altogether, the current knowledge emphasizes the relevance of further characterizing the role of mediators released from peripheral adipose tissue on damage and regenerative failure in both steatotic and nonsteatotic livers undergoing surgery. All of this is required to provide novel therapeutic approaches that can be transferred to clinical liver surgery and consequently increase the number of available donors for transplantation and improve recovery for patients subjected to liver resections.

## 4. Relevance of Adipose Tissue in Patients Undergoing Liver Surgery

Interestingly, the extent of visceral adipose tissue as well as serum levels of adipokines have been evaluated in patients undergoing general surgery. Nevertheless, from our knowledge, pharmacological modulation of adipokine actions has not been reported in the clinical practice of liver surgery [68,69,70,71]. Indeed, the significance of visceral adipose tissue remains controversial in the surgical setting [71].

In liver resections associated with hepatocellular carcinoma (HCC), preoperative visceral adiposity, as well as low muscularity (since obesity might be associated with a decrease in muscle mass), was closely related to postoperative death and HCC recurrence [72,73,74]. In addition, it has been reported that greater fat accumulation in skeletal muscle has been associated with a worse prognosis and survival after PH in patients with HCC, even with adjustment for other known predictors [75]. Moreover, prospective studies and meta-analyses have suggested that obese patients have increased risk and a poorer prognosis for many types of cancer [72,73,74]. All of these results in PH are in line with those observed in living donor liver transplantation (LDLT), since patients with a high degree of muscle steatosis and visceral adiposity show worse survival rates after transplantation compared with patients without obesity or with normal musculature [76]. Nevertheless, these findings are challenged by opposite observations. Indeed, preoperative abdominal computed tomographic (CT) scans in patients undergoing major hepatic resection associated with cancer suggest that obesity does not correlate with poor outcomes after major surgery [77]. Interestingly, neither preoperative visceral adiposity nor low muscularity were poor prognostic factors in patients undergoing liver resection for colorectal liver metastases [78]. In addition, some studies focused on liver resections of different cancer types showed that patients with a higher body mass index (BMI) survive longer than normal-weight patients after surgery [79,80,81,82]. It should be noted that CT measurement enables specific quantification of visceral adipose tissue, which is not reflected by BMI.

The contradictory results in clinical practice, the so-called obesity paradox, might occur due to the different methodologies used to evaluate and measure adipose tissue [71], but different types of surgery (resection vs. transplantation) as well as liver pathologies should also be noted.

Changes in adipokine levels in patients subjected to PH have been reported, suggesting that early-phase elevation of serum levels of hepatocyte growth factor (HGF), leptin, and macrophage colony-stimulating factor (M-CSF) could be associated with the acceleration of liver regeneration [83]. In line with this, plasmatic adipokines after LDLT have been mainly reported as biochemical markers to evaluate the risk of fibrosis progression in patients transplanted due to hepatitis C [84]. However, in these studies, the role of adipose tissue as a source of adipokines was not evaluated.

## 5. Experimental Strategies to Evaluate Adipose Tissue in Liver Surgery

### 5.1. Lipectomy

**Figure 4:**
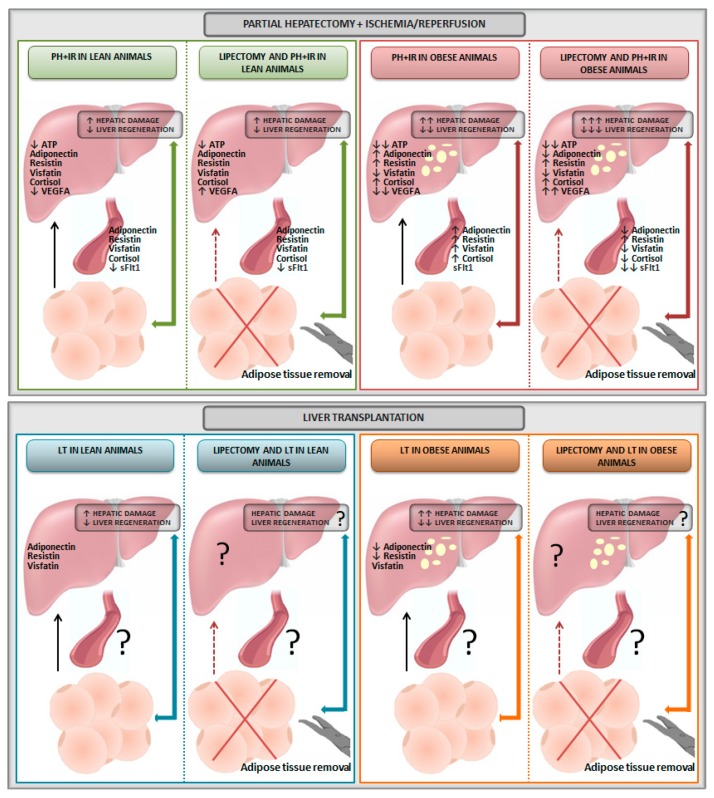
Schematic illustration of lipectomy effects in liver surgery. Abbreviations: ATP, adenosine triphosphate; I/R, ischemia–reperfusion; LT, liver transplantation; PH, partial hepatectomy; sFlt1, soluble form of the VEGF receptor 1; VEGFA, vascular endothelial growth factor type A.

The literature describes the surgical excision of adipose tissue (lipectomy) to evaluate the function of adipose tissue in physiological conditions and different pathologies [37,40,41]. However, few studies have attempted to discern the role of adipose tissue on adipokine levels and hepatic damage and regenerative failure in liver surgery of PH with I/R [56,57,58,59,60], and no studies have evaluated the effects of a lipectomy in livers submitted for transplantation (Figure 4).

The results obtained using lipectomy in PH with I/R indicate that, in contrast with nonsteatotic livers, adipose tissue is required for liver regeneration and to reduce damage in the presence of steatosis [57] (Figure 4). Interestingly, adipose tissue does not seem to be an energy source for the nonsteatotic liver since ATP levels were unchanged after lipectomy [57]. Regarding adipokines, removal of adipose tissue using lipectomy in lean animals undergoing PH with I/R resulted in plasmatic and hepatic levels of adiponectin, resistin, visfatin, and cortisol, similar to those observed under PH with I/R conditions [57,58,59]. The levels of sFlt1 were reduced in plasma in lean animals lipectomized and undergoing PH + I/R, indicating that adipose tissue might be a potential source of sFlt1 [60]. As it has been suggested that circulating VEGFA is sequestered by the sFlt1 released by adipose tissue in lean animals in conditions of PH + I/R [60], this was associated with increases in hepatic VEGFA (Figure 4). In obese animals, the reduced hepatic ATP levels were more evident than in lean animals. However, similar to that occurring in lean animals, adipose tissue does not seem to be an energy source for the steatotic liver, since ATP levels were unchanged after lipectomy. Adipose tissue seems to be an adiponectin source for the steatotic liver, since the induction of lipectomy in obese animals reduced both plasmatic and hepatic levels of adiponectin compared with the results obtained under PH with I/R conditions [57]. The contribution of adipose tissue as a source of resistin was irrelevant since obese animals undergoing PH with I/R showed high levels of plasmatic and hepatic resistin levels, whereas these resistin levels were unaltered under lipectomy conditions [58] (Figure 4). In obese animals undergoing PH with I/R, the visfatin levels were increased and reduced in liver and plasma, respectively. When adipose tissue was removed in obese animals undergoing PH with I/R, circulating visfatin levels were reduced, whereas hepatic visfatin accumulation was unaltered [58]. Thus, PH with I/R induced the release of visfatin from adipose tissue to circulation and reduced the generation of visfatin by the liver [58]. In obese animals undergoing PH with I/R, plasmatic and hepatic cortisol levels were increased [61]. In contrast, lipectomy in obese animals reduced cortisol levels in plasma but not in the steatotic liver [59], although the potential contribution of adipose tissue in the hepatic levels of cortisol of obese animals cannot be discounted. Indeed, changes in the enzymes engaged in cortisol generation and clearance were detected in adipose tissue of obese animals undergoing PH with I/R [59]. The reduced plasmatic levels of sFlt1 in obese animals lipectomized and undergoing PH with I/R were more evident than in lean animals [60]. This increased the circulating VEGFA bioavailability and, consequently, increased the opportunity of VEGFA to be taken up by the steatotic liver [60] (Figure 4).

Reduced adiponectin and resistin levels were observed only in steatotic livers when obese animals were subjected to LT [62] (Figure 4). The role of adipose tissue on hepatic adipokine levels, hepatic damage, and regeneration following LT remains to be elucidated. In our view, given the key role of adipose tissue in steatotic and nonsteatotic livers undergoing PH with I/R, strategies based in the adipose tissue removal should been used to study the crosstalk liver–adipose tissue in LT, mainly in the presence of steatosis. 

### 5.2. Transgenic Animal Models

The use of transgenic animal models has improved our understanding of the pathophysiology of adipose tissue. The main focus of transgenic animal models has been the expression or knockout of selected genes, specifically in adipose tissue, identifying and characterizing promoter regions that confer adipose–tissue specific expression [7]. For instance, to target both white and brown adipose tissue, the promoters for adipocyte lipid binding protein aP215 and for phosphoenolpyruvate carboxykinase are usually used, whereas to target only brown adipose tissue, the mitochondrial uncoupling protein-1 (UCP-1) promoter is used [85,86,87]. In our view, the potential applications of transgenic animal models with overexpression or knockout of adipose tissue-selected genes might be of scientific and clinical interest to evaluate the adipose tissue–liver axis in hepatic resections and transplantation, since in different surgical conditions, hepatic diseases might be improved by directly targeting adipose tissue, rather than liver tissue per se.

## 6. Conclusions

The role of adipose tissue on damage and regenerative failure in experimental liver surgery depends on the type of surgical procedure (PH with or without I/R) as well as the type of liver (steatotic versus nonsteatotic) submitted to liver surgery. This should be taken into account for the establishment of protective strategies modulating the liver–adipose tissue axis, which would be specific for each surgical procedure and type of liver, as it has been reported in the present review. Further clinical studies and appropriate methods for adipose tissue measurement will be required to elucidate the significance of visceral adipose tissue in the clinical scenario of surgical hepatic resections. The use of experimental models of lipectomy as well as transgenic animal models with expression or knockout of adipose tissue-selected genes might be of scientific and clinical relevance to elucidate the contribution of the adipose tissue–liver axis, as well as the role of adipose tissue as an energy substrate and/or a source of different adipokines and hormones in livers subjected to transplantation. This would provide novel therapeutic approaches to be transferred to clinical conditions to improve the post-transplantation outcomes and consequently increase the number of available donors for transplantation.

## Abbreviations

AICAR	Cell-permeable adenosine analog that is a selective activator of AMPK
AMPK	Adenosine monophosphate-activated protein kinase
ATP	Adenosine triphosphate
BMI	Body mass index
BMP	Bone morphogenetic protein
CT	Computed tomography
Fas/CD95	Apoptosis antigen 1 or cluster of differentiation 95
FFA	Free fatty acids
HCC	Hepatocellular carcinoma
HGF	Hepatocyte growth factor
I/R	Ischemia–reperfusion
IL	Interleukin
JNK1	c-Jun N-terminal kinase-1
LDLT	Living donor liver transplantation
LT	Liver transplantation
M-CSF	Macrophage colony-stimulating factor
NO	Nitric oxide
PAI-1	Plasminogen activator inhibitor-1
PC	Preconditioning
PH	Partial hepatectomy
sFlt1	Soluble form of the VEGF receptor 1
SIP1	Smad interacting protein 1
TGF-β	Transforming growth factor-β
TNFα	Tumor necrosis factor α
UCP-1	Mitochondrial uncoupling protein-1
VEGFA	Vascular endothelial growth factor type A
